# Refinement of Sustainable Polybutylene Adipate Terephthalate (PBAT) with Amorphous Hydrogenated Carbon Films (a-C:H) Revealing Film Instabilities Influenced by a Thickness-Dependent Change of sp^2^/sp^3^ Ratio

**DOI:** 10.3390/ma13051077

**Published:** 2020-02-28

**Authors:** Torben Schlebrowski, Halima Acharchi, Barbara Hahn, Stefan Wehner, Christian B. Fischer

**Affiliations:** 1 Department of Physics, University Koblenz-Landau, 56070 Koblenz, Germany; schlebrowski@uni-koblenz.de (T.S.); wehner@uni-koblenz.de (S.W.); 2 Department of Material Analysis, University of Applied Sciences Koblenz, RheinAhr Campus, 53424 Remagen, Germany; halima-87@hotmail.de (H.A.); hahn@hs-koblenz.de (B.H.); 3 Materials Science, Energy and Nano-Engineering Department, Mohammed VI Polytechnic University, Ben Guerir 43150, Morocco

**Keywords:** RF-PECVD, acetylene plasma, synchrotron based surface analytical techniques, chemical composition of uppermost layers, stress release phenomena

## Abstract

The increasing use of polymers is related to a growing disposal problem. Switching to biodegradable polymers such as polybutylene adipate terephthalate (PBAT) is a feasible possibility, but after industrial production of commercially available material PBAT is not suitable for every application. Therefore, surface refinements with amorphous hydrogenated carbon films (a-C:H) produced by plasma-assisted chemical vapor deposition (PE-CVD) changing the top layer characteristics are used. Here, 50 µm-thick PBAT films are coated with a-C:H layers up to 500 nm in 50 nm steps. The top surface sp^2^/sp^3^ bonding ratios are analyzed by X-ray photoelectron spectroscopy (XPS) and near-edge X-ray absorption fine structure (NEXAFS) both synchrotron-based. In addition, measurements using diffuse reflectance infrared Fourier transform spectroscopy (DRIFT) were performed for detailed chemical composition. Surface topography was analyzed by scanning electron microscopy (SEM) and the surface wettability by contact angle measurements. With increasing a-C:H layer thickness not only does the topography change but also the sp^2^ to sp^3^ ratio, which in combination indicates internal stress-induced phenomena. The results obtained provide a more detailed understanding of the mostly inorganic a-C:H coatings on the biodegradable organic polymer PBAT via in situ growth and stepwise height-dependent analysis.

## 1. Introduction

Plastics are now being used in many areas of modern life. Specialized areas of applications are the packaging industry, agriculture and even medical technology. This is made possible by their formability, elasticity, low weight and good chemical resistance [1,2]. Limiting factors of the untreated polymers are their low hardness, low abrasion resistance or poor mechanical properties, which considerably restrict their usability [2]. Another intrinsic problem of common polymers is their poor to impossible compostability and the large amount of waste they generate. This clearly has a negative impact on the environment [3]. Possible alternatives are biodegradable polymers like polybutylene adipate terephthalate (PBAT), which is a flexible, fast-degrading material [4].

In order to further adapt this biopolymeric material to certain applications, they are often improved with amorphous hydrogenated carbon (a-C:H) layers [5,6,7]. The carbon in these layers is π- and σ- hybridized for sp^2^ as well as sp^3^. The sp^2^ clusters are limited to short chains embedded in a sp^3^ matrix containing carbon and hydrogen [5,8]. The properties of the resulting layers strongly depend on this sp^2^ to sp^3^ ratio and hydrogen (H) content. This ratio as well as the H-content can be adjusted by the chosen plasma parameters [5,8,9,10]. Another possibility is the variation of the layer thickness keeping constant plasma parameters [11,12]. Thus, it is possible to obtain the desired layer properties such as high hardness with high electrical resistance (a sp^3^ dominated, diamond-like layer) or a soft, electrically conductive layer (predominantly sp^2^ hybridized carbon, graphite-like layer) [13].

The precursor gas for the plasma operation also contributes to the sp^2^/sp^3^ hybridization ratio of the carbon atoms deposited on the polymer and crosslinked. The change in the hybridization ratio also leads to a chemically different behavior of the resulting layers, which is reflected, for example, in their surface wettability [14]. Thus, the obtained layer can be adapted to the required application by varying the hybridization of the carbon. The most common method for depositing such a-C:H layers is chemical vapor deposition (CVD). This can be additionally supported by the use of high-frequency controlled plasmas (radio frequency plasma-enhanced chemical vapor deposition; RF-PECVD) [5,13,15,16]. The RF-PECVD process has the advantage that it operates at low temperatures and can also coat non-conductive substrates [7,13].

The present study investigates the variation dependence of the carbon hybridization of a:C-H layers by their thickness applied on biodegradable polymer PBAT. The carbonaceous layers were produced by RF-PECVD with acetylene plasma. Ex-situ scanning electron microscopy (SEM) was performed to investigate the surface morphology of these deposited carbon layers. The chemical composition of the resulting layers was analyzed by diffusive reflectance infrared Fourier transform (DRIFT) and the surface-sensitive X-ray techniques near-edge X-ray absorption fine structure (NEXAFS) and X-ray photoelectron spectroscopy (XPS). In addition, contact angle measurements (CA) were performed to evaluate the relationship between the sp^2^/sp^3^ ratio and macroscopic physical aspects.

## 2. Materials and Methods 

### 2.1. Sample Preparation and Deposition of a-C:H Layers

For the coating, films of polybutylene adipate terephthalate (PBAT; industrial quality, Lackner Ventures and Consulting GmbH, Vienna, Austria) were cut into pieces with a size of 10 cm x 10 cm and fixed on vacuum-compatible, self-manufactured aluminum sample holders of similar dimensions and placed in a high-vacuum chamber. The chamber in which the coatings were performed was equipped with a high-frequency (RF, 13.65 MHz) plasma source (Copra DN 400, CCR GmbH, Troisdorf, Germany) [17]. To activate and clean the sample surface, all samples were pretreated with an oxygen plasma (RT, pressure 1 Pa, flux 65 sccm/min, power 200 W, plasma time 10 min) before carbon coating [18,19,20,21]. After oxygen plasma treatment, the PBAT samples were exposed to acetylene plasma (RT, pressure 0.65 Pa, flux 65 sccm/min, power 107 W) [18,19,20,21]. Within these parameters the plasma was operated in H-mode. The temperature of the films never exceeded 40 °C at any time during plasma treatment [21]. By varying the coating time, different a-C:H layer thicknesses of 50–500 nm were realized in 50 nm steps. The deposition rate was about 10 nm/min and is approximately constant over time [21]. A smaller variation was present during the initial growth. During the coating process, the samples were positioned at a distance of 275 mm in front of the plasma source [21], resulting in a r-type diamond-like carbon (DLC) coating [18,19,20]. This is a more hard and flexible coating due to a higher degree of cross-linked sp^3^ carbon centers in the coating caused by a high amount of subplantation processes [21]. In addition, silicon wafers (Silicon Materials, Kaufering, Germany), half covered with aluminia foil, were mounted on the aluminum sample holders, to determine the thickness of the applied layer using a profilometer (Dektak 3, Veeco Instruments Inc., Plainview, NY, USA).

### 2.2. Surface Topography and Wettability

Scanning electron microscopy (SEM515, Phillips, 7 kV, WD 20 mm, FEI Company, Amsterdam, The Netherlands) and contact angle measurements were performed to check the surface condition. In order to avoid charging effects and sample damage due to low conductivity, a ca. 7–10 nm thick gold layer was sputtered onto the samples prior to measurement conducted at least at three different points on the entire sample. This ensured reproducibility and reliability that the data obtained was correct.

Contact angle measurements are a macroscopic technique checking the surface wettability of the various samples. The measurements were carried out using the sessile drop technique on a contact angle goniometer (OCA15EC, Dataphysics Instruments GmbH, Filderstadt, Germany) at room temperature and in ambient air. A dispensing needle deposited a drop of high-performance liquid chromatography (HPLC) grade water (CHEMSOLUTE®, Th. Geyer GmbH & Co. KG, Renningen, Germany) with a volume of 1 µl on the sample surface. Subsequently, the contact angles of the droplets with the surface are measured on the left and right side. Measurements were repeated on at least five different locations on each sample surface to verify homogeneity and for the purpose of averaging.

### 2.3. Chemical Composition

To analyze the binding states and chemical composition of carbon atoms DRIFT, XPS, and NEXAFS measurements were performed. The XPS and NEXAFS data were collected at the beamline HE-SGM at the synchrotron source BESSY II, Helmholtz-Zentrum Berlin, Germany during the low alpha phase. The beamline system and detector are described elsewhere [22]. In addition, the system is equipped with a floodgun to prevent charge induced effects on the sample surface. For the XPS measurements, a full survey spectrum (700 eV–0 eV) was first recorded to determine the total chemical composition. Subsequently, the C1s peak was examined at a minimum of two different sample locations, to analyze the different binding states of the carbon atoms. The set of multiple and repeating measurements was performed to check reproducibility and evaluate the homogeneity and stability of a-C:H layers. Additionally, the O1s peak was measured separately to check its presence or absence. The C1s spectra were analyzed by the commercially available software CasaXPS (software version 2.3.18, Casa Software Ltd., Teignmouth, United Kingdom). Thereby proportions of sp^2^, sp^3^ and C-O bonds in the full C1s peak were identified and evaluated. The results obtained were then plotted with Origin software to the corresponding layer thicknesses.

In NEXAFS investigations, both the C- and O-edge of the samples were recorded. In order to support the correctness of the measurements, the C-edge was recorded also at a minimum of two locations. The NEXAFS measurements were performed in partial electron yield (PEY). Due to the counter voltage, not all electrons emerging from the material reach the measurement electronics and the measurement is more surface sensitive [22]. All the spectra were collected at a photon incidence angle of 55° (‘‘magic angle’’). The spectra C K-edges obtained were evaluated using the commercial software Origin. For this purpose, the spectra were first normalized and then adapted to the decreasing ring current, which is present at the BESSY experimental station during the low alpha phase. Subsequently, a correction of possible contamination of the grid by a previously measured gold edge was carried out. The individual steps are summarized by Watts et al. and described elsewhere [23]. Following this, a further analysis was carried out with a self-written peak evaluation program and plotting by Origin 8.1 software. The O K-edge measurements were only used to verify the presence of oxygen and were not evaluated further.

Further analysis of the chemical composition was performed with DRIFT measurements. A Shimadzu Fourier transform spectrometer (IRPrestige-21, Shimadzu, Kyoto, Japan) equipped with the diffuse reflectance measuring apparatus DRS-8000 (Shimadzu, Kyoto, Japan) was used at ambient conditions [24,25]. Two different spectra were recorded: first, the entire spectrum of 500–4000 cm^−1^ (resolution: 4 wavenumbers, 100 repetitions) to find the focus for the thickness-dependent sections of the spectrum. Second a detailed measurement for the most relevant spectral range of 2800–3100 cm^−1^, the C-H stretching region [16,26]. Here, measurements were performed with a resolution of 1 wavenumber and 300 repetitions. Both measurements were repeated at three different locations to ensure surface homogeneity. The overall reference for a-C:H data analysis and measurements itself was an O_2_ plasma-cleaned PBAT sample. The spectral analysis was done with the commercial IR Solution–FTIR Control Software (software version 1.30, Shimadzu Corporation, Kyoto, Japan). Firstly, multipoint baseline insertion with the integrated manipulation tool followed by a software integrated smoothing manipulation (only changes graph appearance but not the information).

## 3. Results and Discussion

### 3.1. Condition of a-C:H Layers

**Figure 1:**
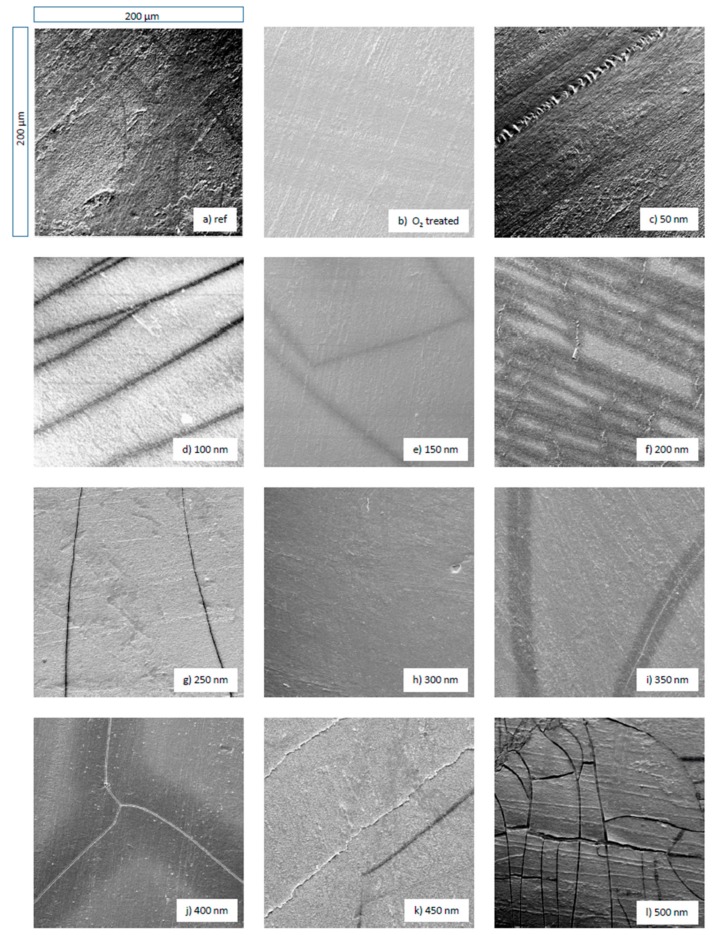
Scanning electron microscope (SEM) images of polybutylene adipate terephthalate (PBAT): (**a**) untreated reference sample, (**b**) treated with O_2_ plasma and (**c**)–(**l**) coated with a-C:H layers. The series from (**c**)–(**l**) shows the changes in topography of an increasing a-C:H coating in 50 nm steps up to 500 nm.

Figure 1 shows the image of pure (Figure 1a), O_2_ plasma treated (Figure 1b) and a–C:H coated PBAT foil samples (Figure 1c–l). The images of the a-C:H coated samples are shown subsequently in ascending order.

A comparison of the O_2_-treated sample (Figure 1b) with the raw polymer (Figure 1a) shows that the previously present impurities have been removed. The surface also appears less roughened and accented. This is mainly due to the sputtering effect by the plasma. The application of O_2_ plasma leads to overall smoothing with only a few particles visible. Possible sources of these particles are remnants from the production and coating processes or dust particles from the ambient air during storage and transport of the films. In addition, parallel, vein-like granulate structures at the surface are visible. These were already existent in the raw polymer, but are now much more pronounced. In parallel, dark, line shaped areas are visible, which cut and interrupt these granulate structures. In the course of the SEM measurements, these lines are present on most images (except for 50, 300 and 500 nm). These are most likely due to a break-up of the gold film applied to the samples for the SEM measurements. 

For the 50 nm a-C:H coated sample there is a closed film on the surface. The granulate structures are emphasized and also the dark areas are visible. This behavior continues up to a layer thickness of 150 nm. The layer remains homogeneous and appears stable. The granulate structures also remain visible. At 200 nm there are smaller fractures in the layer, but these are only locally limited. With increasing layer thickness the layer is still closed and appears homogeneous again. Furthermore, the granulate structures and black areas are present. With a layer thickness of 450 nm the layer starts to crack again until the layer breaks up completely and destructive layer failure occurs at a a-C:H thickness of 500 nm. It is noticeable that the cord buckling effect [27,28] that has been present on polylactide acide (PLA) and polyhydroxybutyrate (PHB) samples [11,12] coated in the same manner as the PBAT samples only occurs to some extent at 450 nm. This is an indication of good adhesion of the layer on the polymer and consequently for a more pronounced interlayer [17,19].

The break-up of the layer at the thicknesses of 200 nm and 450–500 nm can be explained by the residual stress of the coating and substrate combination, which is apparently proportional to the layer thickness. Reasons for the existence of this stress are different coefficients of expansion or material coefficients of both materials (PBAT and a-C:H layer) and processes during the coating like the so-called island formation of the a-C:H layer and an interaction of these islands with each other in early growth phases [29]. If the residual stress exceeds a critical value, the layer begins to break [5]. Up to this point, the layer on the polymer is stable and adheres well without the presence of the cord-buckling effects (only rudimentarily at 450 nm). 

This results in layer thicknesses at which the layer adheres stably to the PBAT after completion of the coating process. Film thickness below 400 nm provides homogeneous and closed layers. An exception is the layer for 200 nm, at which slight local layer failures occur. Higher layer thicknesses lead to a stress-induced layer fracture of the applied layers. Whether closed, homogeneous layers can be restored with increasing thickness and this must be clarified with further measurement campaigns.

Three factors determine the surface wettability and thus the resulting contact angle for the a-C:H coatings investigated here: the morphology of the surface [30,31,32], the existing chemical bonds [33,34,35,36], and various hybridization states of carbon on the surface [10,37,38]. Consequently, contact angle measurements provide information on the structural and chemical properties of the current surface. For example, an oxidized surface leads to an increased attractiveness of water due to the formation of oxygenated groups [36]. However, if the free bonds on the surface are saturated with hydrogen, the strong C–H bonds prevent further interaction between the water applied for the measurement and the sample surface [33,34,35]. The behavior is more hydrophobic compared to a less hydrogenated surface. Various carbon hybridization states also lead to a change in the contact angle behavior at the sample surface [10,37,38]. For a sp^3^ dominated surface, the surface energy is very high due to its strongly covalent character compared to a sp^2^ wetted surface, which has a low polarity of its bonds [10,13]. This leads to a reduced contact angle for sp^3^ rich surfaces, since an increase in polarity results in smaller contact angles and higher hydrophilicity [39].

**Figure 2:**
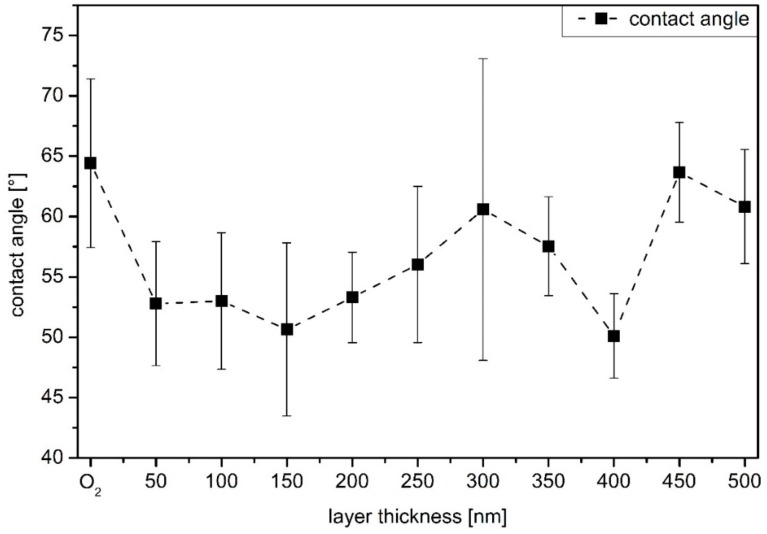
Contact angle results for the O_2_ plasma cleaned and a-C:H coatings on PBAT previously treated by O_2_ plasma with increasing thickness (the dashed line only indicates a trend of the values).

In Figure 2 the contact angle results for PBAT samples coated with a-C:H layers of different thickness are displayed. The reference measurement is the O_2_ plasma-treated sample, since all samples were treated with O_2_ plasma prior to the carbon coating. Overall, the error bars obtained during the measurements are relatively large due to the original wavy structure of the PBAT films. Due to this wave structure, the contact angles can only be evaluated with regard to the chemical structure, as measurements of surface roughness are not possible. However, a trend can be identified: With the application of a 50 nm a-C:H layer, the contact angle drops from 64° to 52° indicating a higher proportion of sp^3^ bonds. With increasing film thickness the contact angles are in an interval of 51–53°, which keeps constant up to 200 nm. From 250 nm on, the contact angle increases steadily until it reaches a maximum at 300 nm. This change in the contact angle is accompanied by a change in the surface free energy, which is explained by different chemical structures and bonds as discussed before. The proportion of sp^2^ bonds increases at the expense of the sp^3^ ones and leads to an increasing contact angle. Up to a layer thickness of 350 nm a-C:H, the values of the contact angles are between 56° and 61° until they drop down to a value of 50° at the 400 nm layer. A renewed restructuring of the carbon network towards sp^3^-dominated surfaces is responsible for this, which is also confirmed later by XPS measurements. With a further increase to 450 and 500 nm, the contact angle quickly increases again to 64° and 61°, respectively. In this region, a layer failure also occurs in accordance with the SEM measurements (compare Figure 1k,l).

### 3.2. Chemical Composition of the Sample Surface

**Figure 3:**
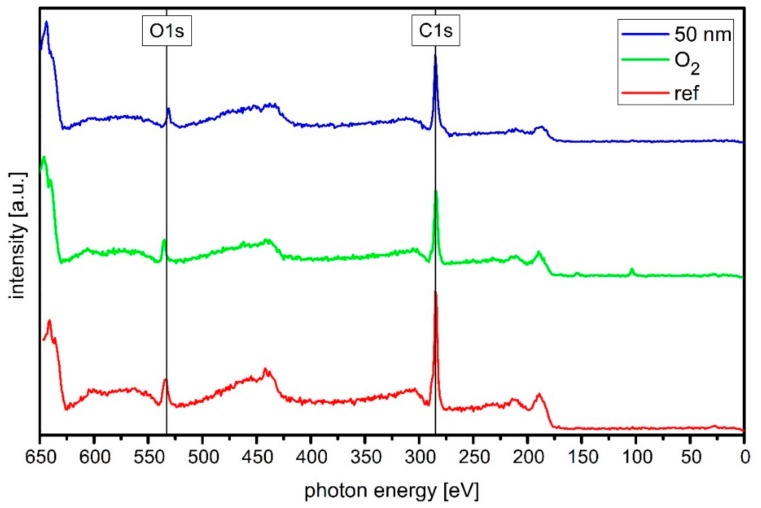
Full X-ray photoelectron spectroscopy (XPS) spectra of raw (red), O_2_ treated (green) and 50 nm a-C:H layer coated PBAT sample (blue). The intensity variation of the oxygen peak as well as the shape of the C1s peak significantly changes as the treatment of the polymer surface progresses.

XPS measurements were performed to analyze the chemical composition and especially the carbon hybridization of the sample surface. Figure 3 shows the results of the XPS measurements for a raw PBAT sample, one treated with the O_2_ plasma and an exemplary one with a 50 nm a-C:H coating. The data analysis was carried out as described in Section 2. The peak positions were determined through the NIST database and compared to literature [40,41,42].

First, the untreated PBAT reference sample (red) is applied. If this sample is treated with an O_2_ plasma (green), the shape of the C1s peak changes (285 eV). The peak side becomes more symmetrical and less pronounced, which indicates reduced C–O bondings. This bond is also superimposed within the C1s peak. It is noticeable that the C KLL peaks (~440 eV) disappear with the O_2_ plasma treatment. If a 50 nm a-C:H layer (blue) is applied to the previously O_2_ treated sample, the acetylene plasma species will react with the prior activated O1s (approx. 533 eV) bonds present on the sample. This initiates and forms the a-C:H layer. The O1s peak is reduced in the XPS survey compared to the O_2_ plasma-treated sample. In addition, the C1s peak has become more symmetrical and the C–O peak it contains has almost disappeared. Both plasma treatments thus cause a significant change in the shape of the C1s peak.

**Figure 4:**
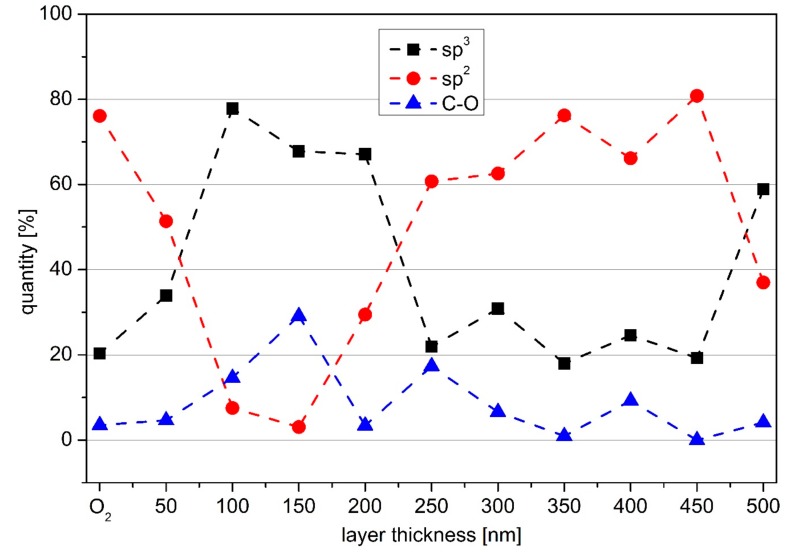
Results for the deconvoluted X-ray photoelectron spectroscopy (XPS) spectra for PBAT. The percentages for the amount of sp^3^ (squares), sp^2^ (circles), and C–O bond (triangles) for coatings with increasing a-C:H layer thickness are shown (dashed lines are only included for clarity).

Figure 4 shows the XPS results for the series of a-C:H layers applied to the previously O_2_ plasma-treated PBAT, which is also included as reference. The percentages of the sp^2^ and sp^3^ bonds in the C1s peak were determined from XPS and their quantity plotted according to the layer thickness. For the evaluation of the data the C–O and the C=O peak were determined separately, but were plotted and discussed together as C–O in Figure 4. The individual deconvoluted spectra of the C1s peaks are attached as Supplementary Materials. In the range of 50–200 nm the sp^3^ bond is dominant. An initially sp^3^-dominated growth of the a-C:H layer on polymer samples has already been observed on other materials such as PLA and PHB [11,12]. The dominance of the sp^3^ bond is in line with the contact angle measurements. With a layer thickness of 250 nm, the dominant bond changes from sp^3^ to sp^2^. This change has also been observed for other materials, even if the turning point varies depending on the material. If the layer thickness continues to increase, the proportion of sp^2^ bonds in the uppermost layers of the film also increases. The system achieves a maximum amount of sp^2^ bonds at 450 nm. From 500 nm, the sp^3^ bonds increase again and dominate. The C–O bond is variable in the first 300 nm, and reaches a maximum at 150 nm to decrease thereafter again. From 300 nm it remains more or less constantly low below 5 %. 

A comparison of the results from the XPS with those from the SEM shows that the changes in the dominant bond are related to failures in the applied a-C:H layers. As the sp^3^ content of the carbon bonds increases, the rigid network of sp^3^ bonds creates stress in the layer and increases together with the bond content [43,44]. C. A. Davis introduced a model for stress generation in such layers [29]. One way to relax the network is to introduce energy through thermal spikes into the network [29]. The other possibility is to break the bonds by too much stress. The layer failure that occurs in the SEM images is most likely caused by such bond break-up. The offset between layer failure and maximum sp^3^ content could be due to a good bond between polymer/layer and a resulting delay until the whole network is riddled with such cracks and the break is visible. The extremely high sp^3^ bond content could also be responsible for the high oxygen content in the 100–200 nm layer thickness range. Immediately after coating, the vacuum chamber was vented with ambient air. The high sp^3^ content on the surface implies a high degree of stress. Due to the mechanical stress during removal of the samples or due to the time needed for discharge of the stress during storage/transport, the bonds on the surface can be broken. As this happens in ambient air, the oxygen contained in the sample can occupy the now free dangling bonds and as a consequence a high proportion of oxygen is obtained in the following ex situ surface-sensitive measurements. The relaxation of the network as presented by Davis [29] combined with a stress release due to layer failure could be responsible for a rising sp^2^ content after a layer thickness of 200 nm. For the layer failure at 500 nm no oxygen saturation takes place. With an increase of the layer from 200 to 250 nm, the sp^3^/sp^2^ ratio drops to a value below one. At a layer thickness of 200 nm, the sp^3^ is dominant. This changes around 250 nm and the sp^2^ one dominates accompanied by the occurrence of slight breaks and minor cracks, but the applied layer remains stable on the sample. 

The second change in dominant bonds takes place from 450 to 500 nm. Here the sp^3^ alternates with the sp^2^ as the dominant bond. Once again, this is accompanied by a layer failure, this time right at the change of the bond ratio. At a layer thickness of 500 nm, a strong layer failure is clearly visible. It is noticeable that the layer does not detach from the substrate and rolls up, but remains firmly attached to it. This is an indication of an accentuated interlayer between substrate and a-C:H layer, so that the layer stays on the substrate despite layer failure. The XPS measurements show no change in the oxygen content of the sample, so no oxygen saturation takes place. A possible explanation is the time when the layer failure occurs on the samples. If the stress in the layer is that high, and layer failure takes place during the plasma process, the free bonds are probably saturated by hydrogen or carbon again, as oxygen is not available here. If the stress only leads to coating failure during ventilation or the mechanical load during sampling, the sample is already under ambient air and oxygen can occupy the now free bonds. Up to a layer thickness of 350 nm, the results of the contact angle are in line to the XPS results. However, it would be expected that the contact angles would remain strong up to a value of 450 nm and then drop rapidly, as the sp^3^ content from here on is much higher than before. The reason for this difference cannot be stated precisely, but the layer failure (increased surface roughness) and the wavy structure of the original PBAT film are reasonable possibilities.

The results found here only partly correspond to those of a-C:H layers on both biopolymers PLA and PHB [11,12], which were coated simultaneously in the same process. Here the layer failure is a consequence of the change from sp^3^ to sp^2^ as dominant bond. The change from sp^2^ to sp^3^ has no influence. In addition, the layer breaks only after the change of binding, so that the change of binding is an indication for an early layer failure. In the case of PBAT, however, there is a small layer failure followed by a change of the dominant binding from sp^3^ to sp^2^, so that the layer failure is an indication for the binding change. The change from sp^2^ to sp^3^ as dominant bond, as it is present at 450 nm layer thickness, leads to a strong layer failure. For other materials, this direction of change for the dominant bond was without consequence [11,12], so it could be additionally a substrate effect. It is possible that the layer failure at 450 nm is due to the bond change from sp^3^ to sp^2^ at 250 nm, only that it occurs with a strong delay of 200 nm layer thickness. Similar findings have already been made for PHB [12]. However, the break-up of the layer at 200 nm would then also be due to a late reaction of the layer to such a change. For this purpose, the layer thickness range of 10–50 nm would have to be examined more closely to determine whether such a change of a dominant bond is present and if an interlayer can be located [45].

**Figure 5:**
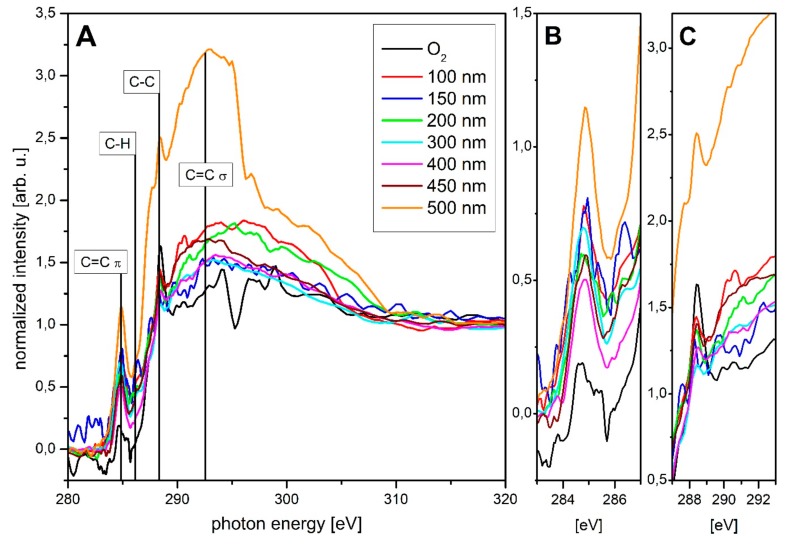
C K-edge near-edge X-ray absorption fine structure (NEXAFS) spectra of an O_2_ treated PBAT sample as well as a-C:H coated PBAT with presented layer thicknesses of 100-500 nm in 100 nm steps. (**A**) displays full spectra, (**B**,**C**) zooms for 283 eV to 287 eV and 287 eV to 293 eV.

**Figure 6:**
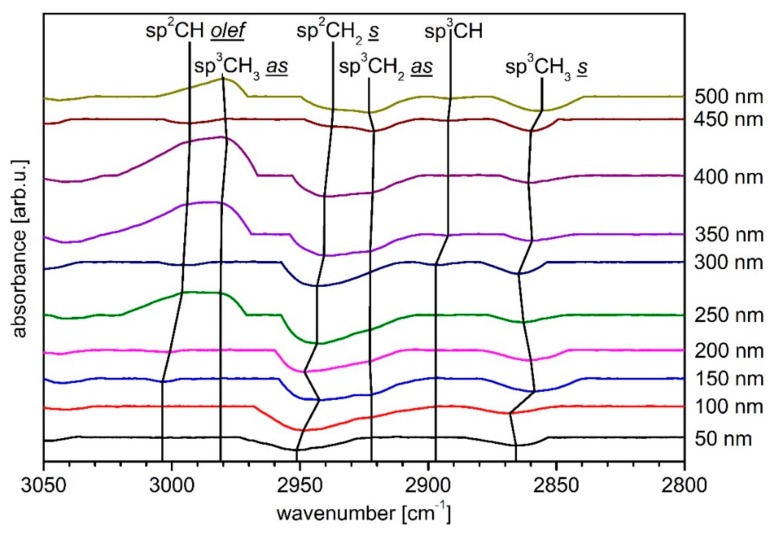
Diffuse reflectance infrared Fourier transform spectroscopy (DRIFT) spectra of the analyzed a-C:H layers with increasing thickness on PBAT. The rising layer thickness is indicated by changing colors with number labelling right next to it.

Figure 5 shows the spectra of the C K-edge NEXAFS analysis. For a better overview, only the layer thicknesses of 0–500 nm are applied in 100 nm steps. Exceptions are the layer thicknesses of 150 nm and 450 nm. An overview of all NEXAFS C K-edge spectra is given in Supplementary Materials. The analysis was performed as described in the experimental details. The positions of the peaks analyzed here were determined as follows: C=C π (284.85 eV), C–H (286.15 eV), C–C (288.35 eV) and C=C σ (292.55 eV) [46,47,48,49,50]. Although the region from 290 to 310 eV also represents the C–C bond, only the peak at 288.35 eV was specifically discussed, as this is the most pronounced for this bond type. The peak also changes with closed, thick layers, so it is not (only) due to the basic PBAT structure. Considering the penetration depth of the very surface-sensitive PEY NEXAFS measurement, changes here are only due to changes in the chemical composition of the layer. A thickness-dependent peak change has already been observed and discussed for other polymers (e.g., PLA, PHB), even if a substrate effect is visible [11,12]. In the following, these peaks and their course will be examined in detail. The application of an a-C:H layer up to 100 nm thickness shows the increase of the C–C peak. The peak for the C=C π binding increases, while the C=C σ binding is less pronounced and almost disappears as the surrounding signals increase. However, its height remains almost unchanged and it broadens a little. Overall, the peak is therefore stronger. An existing C–H peak remains weak but constant. With the application of a 150 nm layer, the C–C peak, which represents the sp^3^ bond, decreases while the C=C π bond becomes more dominant. This is consistent with the results obtained in XPS, where a decrease of the sp^3^ and an increase of the sp^2^ content occurs at this layer thickness and the C–H peak becomes more pronounced. With a layer thickness of 200 nm, the peak of the C–C bond grows again, while the C=C π peak weakens again. The C–H peak and the C=C σ peak are emphasized more strongly again, the surrounding signals of the C=C σ peak decrease and the peak becomes more narrow. The C=C σ peak thus decreases just like the C=C π peak. When thickness exceeds, the C–C peak decreases rapidly and the C=C π peak increases until it begins to weaken again after reaching a layer thickness of 400 nm. This behavior continues with increasing layer thickness. With a thickness of 450 nm, the C–C peak increases again and becomes more accentuated. The other peaks (C=C σ and C–H) are very weak and undefined over the entire measurements. Thus, the measurement data obtained in NEXAFS are in accordance with those obtained in XPS over wide ranges and thus confirm these measurements. Differences in the results of the respective measuring methods are, however, clearly visible in some places and probably partly traceable to the different penetration depths, especially since the NEXAFS measurements are very surface sensitive due to the use of PEY and the XPS measurements that have a comparatively higher penetration depth. The XPS measurements were performed at a larger number of spots than the NEXAFS measurements due to the shorter duration of individual measurements and provide the same results over the sample surface. The NEXAFS measurements themselves also provide similar results at different measuring points. Differences in the respective results are, therefore, not due to singular events on the surface, but rather to the different penetration depths already mentioned. In order to analyze the chemical structure of the a-C:H layers more precisely, DRIFT measurements were performed. For the analysis of the films, an O_2_-treated sample was taken as reference, since all a-C:H coated samples were previously treated with O_2_ plasma. The spectra obtained with DRIFT were evaluated on the basis of infrared spectroscopy [26] and previous results [16,19,20,51,52]. The following steps were performed: first a full overview (450–4100 cm^−1^) was taken to check in which regions the samples differ. Subsequently, detailed measurements of the C–H stretching region, which is in the range 2800–3050 cm^−1^, were arranged to allocate the =CH_x_ and –CH_x_ bonds [16,26,51,52]. The spectra obtained during these measurements are plotted in Figure 6 in ascending order according to the thickness of the layer.

The sample coated with 50 nm a-C:H has only two absorption peaks. Firstly, the sp^2^CH_2_ symmetrical peak at a wavenumber of 2951 cm^−1^ and the sp^3^CH_3_ symmetrical peak at 2866 cm^−1^ [51]. Both peaks are clearly visible. The formation of new and shifting CH_2_ groups is a strong indication for an interlayer formation [19,20]. The interlayer formation can be explained by three dominant subplantation processes [53]. A non-specific etching of the entire surface area due to the several plasma species, the insertion of C and H^+^-ions into the sample, and the adsorption of plasma radical species to form new bonds on the surface. According to the latest results of Catena et al. for comparable polymer substrates, the interlayer formation process ends up at a layer thickness of around 50 nm [19,20]. If the layer thickness increases to 100 nm, a third peak appears at a wavenumber of 2920 cm^−1^. This peak can be assigned to the asymmetric sp^3^CH_2_ [51]. This peak is, notwithstanding, only weakly formed. The other two peaks are retained. The sp^2^CH_2_ symmetric peak undergoes a shift to lower wavenumbers with increasing layer thickness, which indicates a changing distance of the C–H bond [26]. This changing bond length is already known for a-C:H layers on other polymers [11,12,18,20]. If the layer thickness rises to 150 nm, a small peak appears at 3003 cm^−1^ which can be associated with the sp^2^CH olefins [52]. A layer thickness of 200 nm results in a short backshift, which is reversed with increasing layer thickness.

If the layer thickness continues to increase, the sp^2^CH_2_ symmetrical peak is increasingly emphasized. At 250 nm, this peak is very pronounced. Here, two further peaks become visible, but they overlap each other. In addition, these peaks are formed in such a way that these bonds are less present compared to the O_2_ plasma-treated PBAT sample. They are 2980 cm^−1^ and 2995 cm^−1^ and are linked to the sp^3^CH_3_ asymmetric [51] bond and the shifted sp^2^CH olefin bond [52]. The reduction in sp^3^ bonds corresponds to the results obtained in XPS. When a layer thickness of 300 nm is reached, the peaks appearing at 250 nm almost disappear again. Only the sp^2^CH olefin peak is weakly visible, this time as absorption peak. In addition, a peak at 2897 cm^−1^ is visible, which can be assigned to the sp^3^CH bond [52]. With a 350 nm a-C:H layer, the sp^3^CH_3_ appears asymmetrically and the sp^2^CH olefin peak again, but again not as absorption peak, and the sp^3^CH_2_ asymmetric peak appears again. Both the sp^3^CH peak and the sp^3^CH_3_ symmetric peak shift to smaller wavenumbers. It is noticeable that the peaks belonging to the sp^3^ bonds are weaker here. This behavior continues with increasing layer thickness and is consistent with the XPS measurements, which predict the lowest fraction of sp^3^ bonds here.

With a layer thickness of 450 nm, the sp^3^CH_3_ symmetric peak is more pronounced again together with the sp^3^CH_2_ as peak. The overlapping sp^2^CH olefin and sp^3^CH_3_ asymmetric peaks become here absorption peaks. In addition, the sp^2^CH_2_ symmetric peak is only weakly emphasized. This also corresponds to the XPS measurements, which predict a reduction of the sp^2^ and an increase of the sp^3^ content at this layer thickness. At 500 nm the sp^3^ bonds are even stronger. It should be mentioned that the sp^3^CH_3_ symmetric peak shifted to lower wavenumbers and the sp^3^CH_2_ asymmetric peak to higher. While the sp^3^CH_3_ symmetric peak continues to strengthen, the sp^3^CH_3_ asymmetric peak also becomes visible again. However, its orientation once again indicates a weakening of this bond compared to the O_2_ plasma-treated sample. Altogether, the strengthening of the sp^3^ bonds is again consistent with the results obtained in XPS, which predict the sp^3^ bond as the dominant ones. The peaks belonging to the sp^2^ bonds have almost disappeared here.

In summary, the results obtained with DRIFT support those from XPS and NEXAFS. In addition, the DRIFT measurements provide more detailed information on how precisely the carbons are bound. A large number of shifts in the wavenumbers associated with the bond lengths show a steady change in the chemical bond ratios when depositing the a-C:H layers on PBAT, which depends on the thicknesses of the applied layers.

## 4. Conclusions

Using a RF-PECVD process and acetylene as precursor gas, the biodegradable polymer PBAT was coated with a-C:H layers of different thicknesses. The resulting samples were analyzed with SEM, by contact angle measurements and, furthermore, DRIFT spectroscopy, NEXAFS and XPS in order to establish their chemical composition. SEM analysis revealed that stable a-C:H layers can be achieved on PBAT if the thickness does not exceed 200 nm. Thereafter, the dominating bond situation changes from sp^3^ to sp^2^. The layer is ruptured by the high stresses in the layer associated with the emerging sp^3^ bond. The damage itself seems to be only superficial and insignificant. Also in the DRIFT measurements a sp^2^ peak is formed at 250 nm, which supports the change from sp^3^ again to sp^2^ as dominant bond. Supporting NEXAFS and XPS data additionally confirm the changing chemical composition of the layer with increasing thickness. The DRIFT investigation is underlined by the fact that a large number of shifts in wavenumbers belonging to different bonds correspond to fluctuating bond lengths and, therefore, reveal a changing chemical environment in the a-C:H layers. In the further course, the layer growth remains stable until the layer thickness reaches 450 nm. At this point the dominant bond also changes, this time from sp^2^ to more sp^3^. 

In summary, the deposition of a stable a:C-H layer on biodegradable PBAT is possible up to a thickness of 150 nm and for the range between 250 nm to at least 400 nm. However, complete delamination of the film does not occur. Despite some tearing, the layer remains adherent to the polymer. A comprehensive analysis of the chemical composition by using synchrotron-based XPS and NEXAFS in combination with DRIFT was performed for the present carbon layers and underlines a significant changing character for the layers with increasing deposition height. Apart from minor deviations, the results of the three techniques used for the chemical analysis of the coating are consistent. The stable adhesion of the a-C:H layers despite breakage of the layer underlines the formation of a distinct interlayer between the polymer and the a-C:H layer.

The analyses carried out and presented here show that the bonding ratios of the carbon atoms depend not only on the plasma parameters selected, but also on the layer thickness achieved. It follows that it is also possible to adapt the layer obtained to the selected applications (e.g., hydrophilic, -phobic) by monitoring the layer thickness. This is possible because there is a layer thickness-dependent change between the sp^3^- or sp^2^-bound dominant carbon types in the present a-C:H coating process on PBAT. Since the properties of the applied layer also change as a result of the change in the bonding ratio, these can be controlled accordingly in this way.

## Supplementary Materials

The following are available online at https://www.mdpi.com/1996-1944/13/5/1077/s1.
