# Supplementary figures and images for: Refinement of Sustainable Polybutylene Adipate Terephthalate (PBAT) with Amorphous Hydrogenated Carbon Films (a-C:H) Revealing Film Instabilities Influenced by a Thickness-Dependent Change of sp2/sp3 Ratio
Source: Materials (Basel). 2020 Feb 28;13(5):1077. doi: 10.3390/ma13051077 (PMC7084182; doi:10.3390/ma13051077)

O<sub>2</sub>

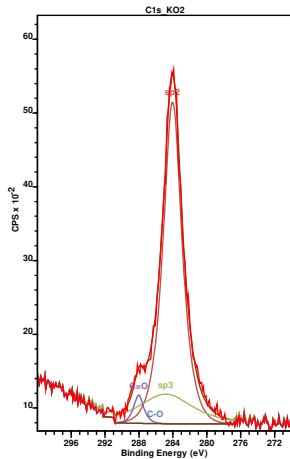

200 nm

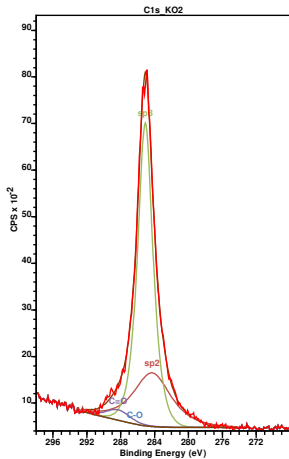

400 nm

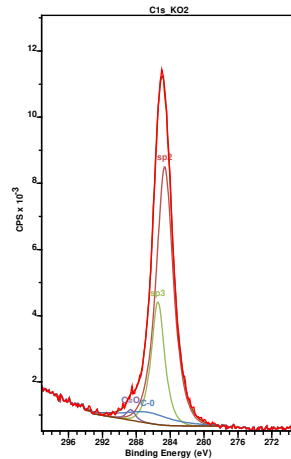

50 nm

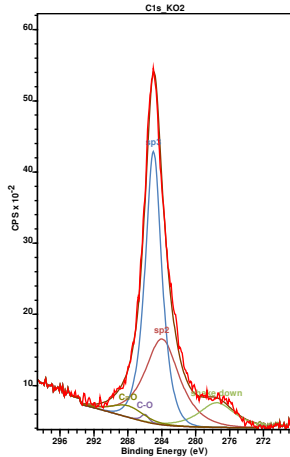

250 nm

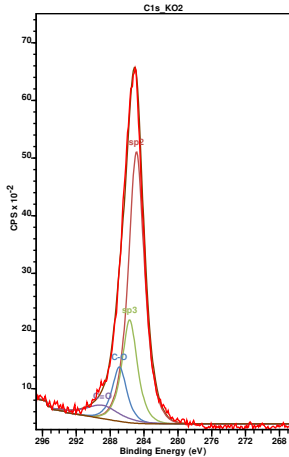

450 nm

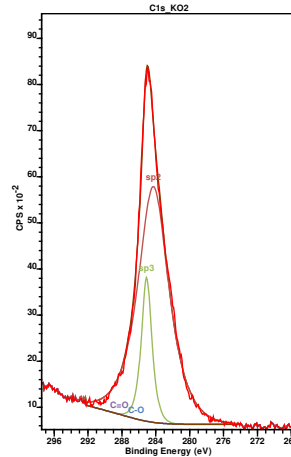

100 nm

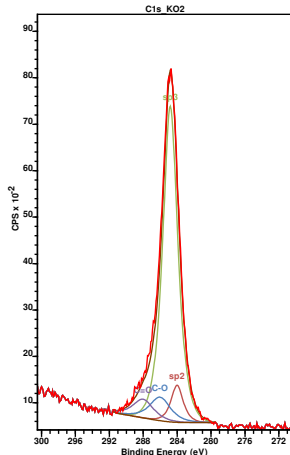

300 nm

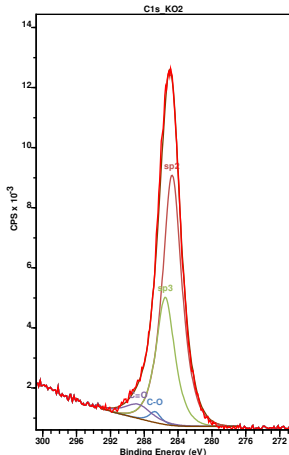

500 nm

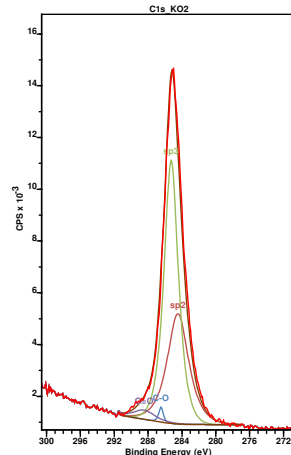

150 nm

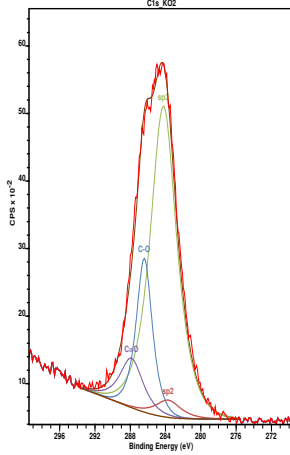

350 nm

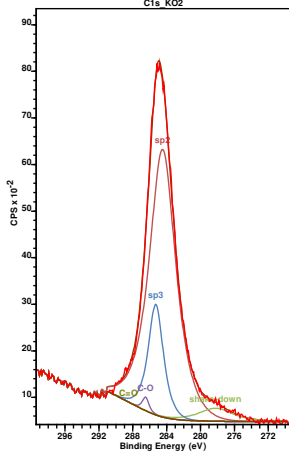

# NEXAFS

## C K-edges

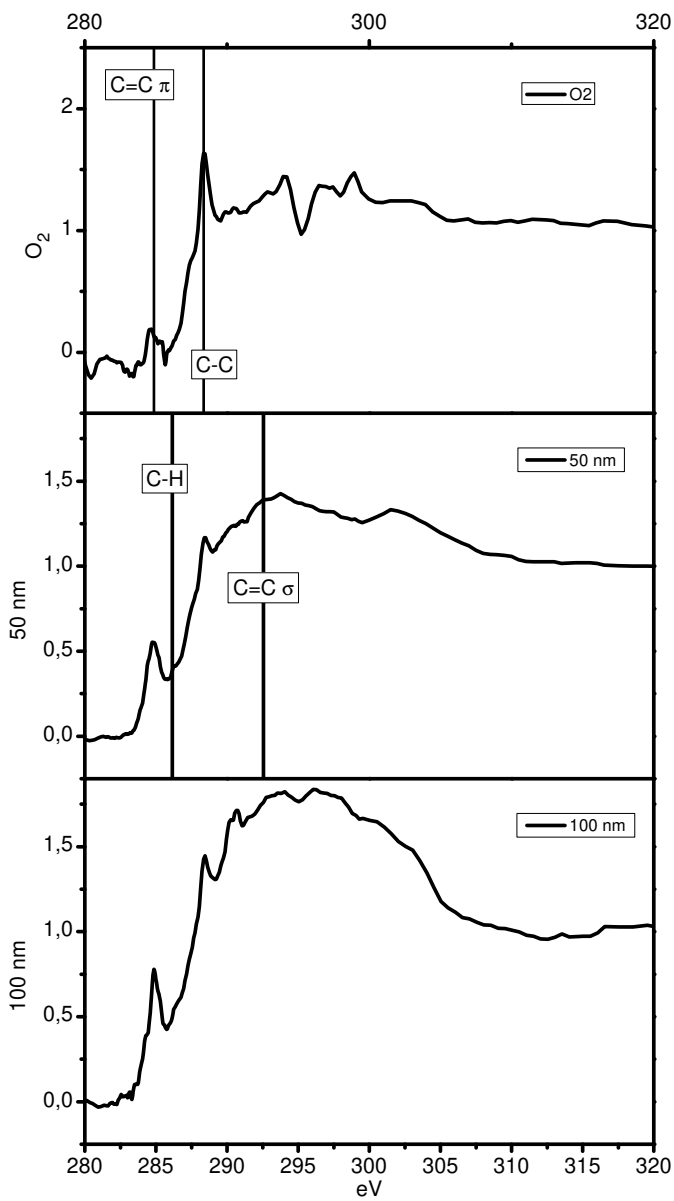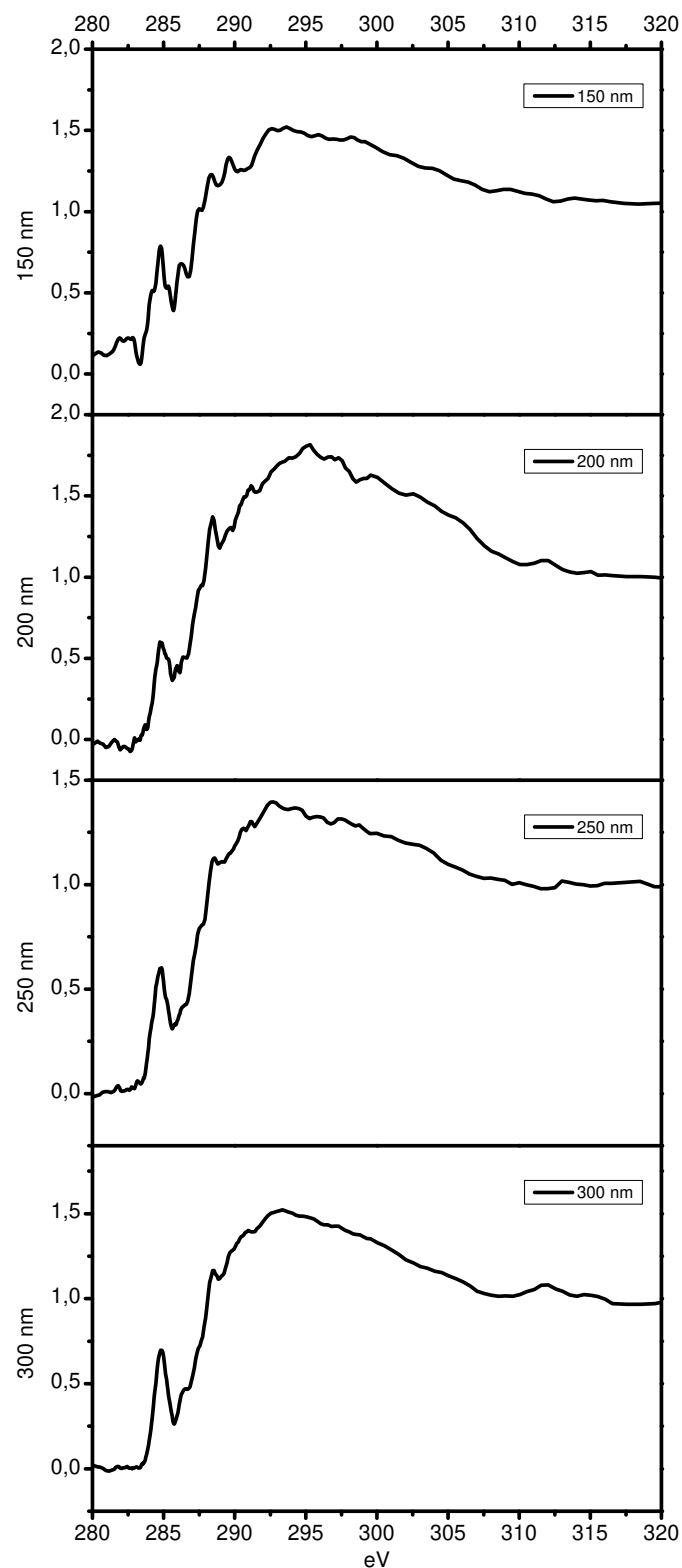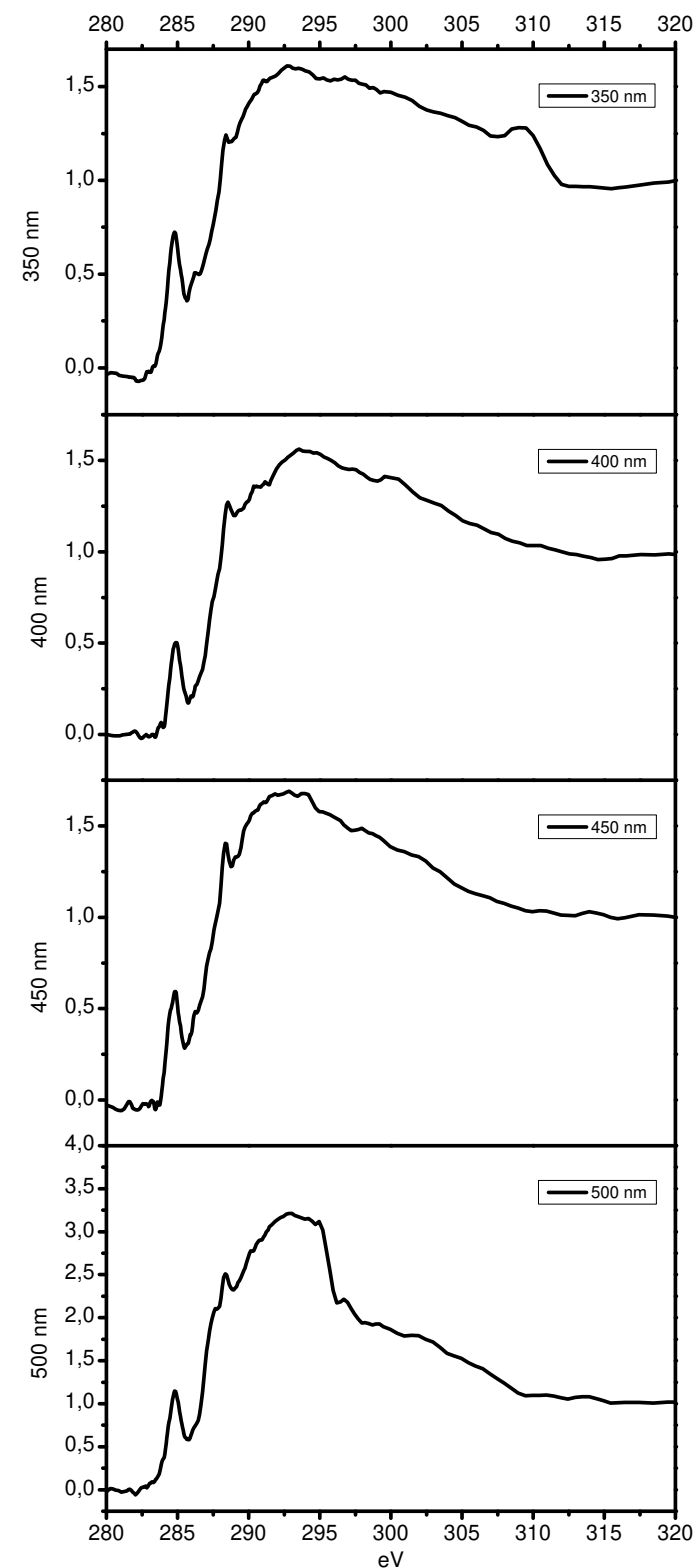

Supplement: Supplementary file 1 [file materials-13-01077-s001.pdf]
